# Synthesis of Thiophene and NO-Curcuminoids for Antiinflammatory and Anti-Cancer Activities

**DOI:** 10.3390/molecules18021483

**Published:** 2013-01-25

**Authors:** Mahera M. Ahmed, M. Akram Khan, Kim Drummond Rainsford

**Affiliations:** Biomedical Research Centre, Sheffield Hallam University, Howard Street, Sheffield S11WB, UK

**Keywords:** NO-NSAIDs, synthesis, curcuminoids, cytotoxicity, cytokines, chemokines, anti-cancer

## Abstract

In search of better NSAIDs four novel nitric oxide donating derivatives of curcumin (compounds **9a**–**d**), and four thiophene curcuminoids (compounds **10a**–**c**, **11**) have been synthesised. The cytotoxic effects of these compounds along with the lead compound curcumin (**7**) and their effect on the production of the reactive oxygen species nitric oxide and pro-inflammatory cytokines IL-1β, TNF-α and chemokine CXCL-8 were evaluated using human monocytic THP-1 and colon adenocarcinoma CACO-2 cell lines. All of the nitric oxide donating curcuminoids **9a**–**d** and the thiophene curcuminoids **10a**–**c** and **11** were non-cytotoxic to THP-1 cells over a concentration range of 10-100 μM and compared with curcumin compounds **10b** and **10c**, were more toxic. In CACO-2 cells, **10b** and **11** appeared to be non-toxic at 10 to 50 μM, whereas **10a** and **10c** were non-cytotoxic at 10 μM only. These results clearly indicate that the introduction of a nitroxybutyl moiety to curcumin and replacement of phenyl rings with thiophene units reduces the cytotoxic effect of the parent curcumin, whereas a methyl substituted thiophene increases the cytotoxic effects. In THP-1 cells, drugs **10a** and **11** significantly decreased IL-1-β production at their non-cytotoxic concentrations, whereas, they did not decrease TNF-α production in CACO-2 cells. Compound **11** showed a significant decrease in CXCL-8 production.

## 1. Introduction

In the last two decades curcumin and its derivatives have received widespread interest for their therapeutic effects for various indications [1] for example, in treating inflammatory conditions and cancer [1,2,3,4,5,6,7]. We recently reported the anti-inflammatory activities, determined *in vivo* and *in vitro*, for a range of novel aromatic and heterocyclic aromatic curcuminoids [8]. Some of our novel curcuminoids may form the basis for development of new low gastro-toxicity anti-inflammatory agents with selective activity as inhibitors of cytokine inflammatory mediators. 

**Figure 1:**
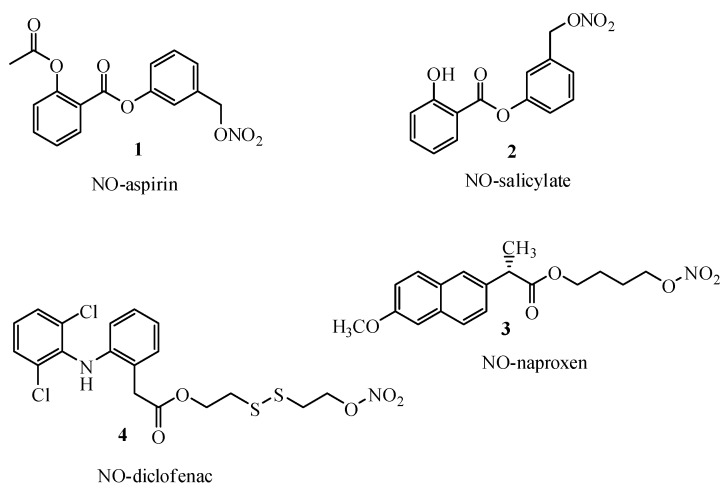
NO-NSAIDs reported in the literature.

Here we report the anti-inflammatory properties of some nitric oxide donating curcuminoids **9a**–**d** analogous to the nitric oxide donating non-steroidal anti-inflammatory drugs NO-NSAIDs. Nitrate esters such as cellulose nitrate and glycerol nitrate are esters of nitric acid that have been known for over 150 years. However, recently the synthesis of nitrate esters and in particular 4-nitroxy-1-butylcarboxylates has received a renewed interest as antiinflammatory agents [9,10,11,12,13,14]. The development of NO-NSAIDs is based on various findings whereby NO compliments some of the properties of the prostaglandins (PGs) within the gastric mucosa, thus the NO-coupled NSAIDs might deliver NO to the site where NSAIDs induce micosal damage, thereby decreasing the gastric toxicity, which is often a consequence of diminished PG levels in the gastric mucosa [15]. NO-aspirin and other NSAIDs **1**–**4** with NO donating activity have been reported to suppress platelet aggregation and thereby inflammation with much reduced gastrointestinal tract injury. These NO-NSAIDs (Figure 1) are reported as stable in aqueous solutions and well absorbed after oral administration [15]. Though a number of studies have shown NO-NSAIDs to have enhanced anti-inflammatory activity with lesser side-effects, their major limitation is the rapid release of NO with relatively small quantities being produced in the systemic circulation [16,17,18]. The NO donating curcuminoids **6**–**9** were developed seeking potentially prolonged production of NO *in vivo*.

**Figure 2:**
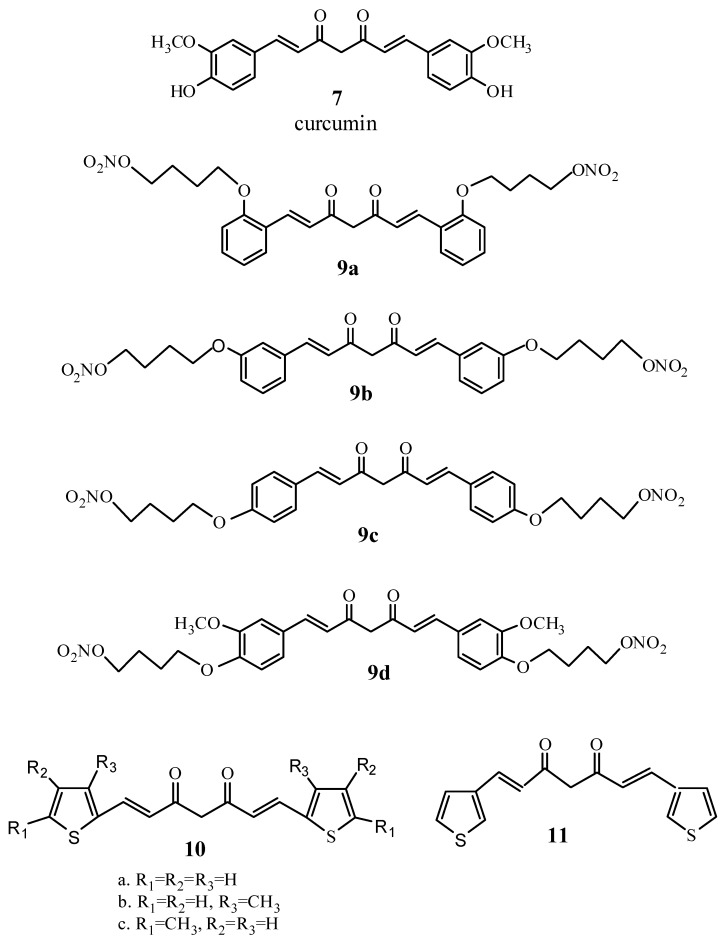
Synthesised thiophene and NO-curcuminoids.

Here we report the synthesis of NO-curcuminoids **9a**–**d**, all containing the 4-nitroxybut-1-yl group as a nitric oxide donating group. Their cytotoxicity and potential anti-inflammatory and anti-cancer activities are reported in terms of effects on NO, the cytokines IL-1, TNF-α and chemokine CXCL-8 (IL-8) production relative to the naturally occurring curcumin (**7**). Some novel thiophenyl curcuminoids **10a**–**c**, **11** [8] (Figure 2) were also investigated in human monocytic THP-1 and adenocarcinoma-derived CACO-2 cell lines. The effects on the cytokines were studied as chemokine CXCL-8 has similarly been reported to be involved in the pathogenesis of a number of disorders including inflammatory bowel diseases [19,20,21]. The cytotoxic effects of thiophene derivatives **10a**–**c**, **11** complexed with HP-γ-CD were also assessed using the THP-1 cells. IL-1β and TNF-α, initiates a cascade of events leading to inflammation and tissue destruction in various inflammatory diseases [22,23,24].

## 2. Results and Discussion

### 2.1. Chemistry

**Scheme 1:**
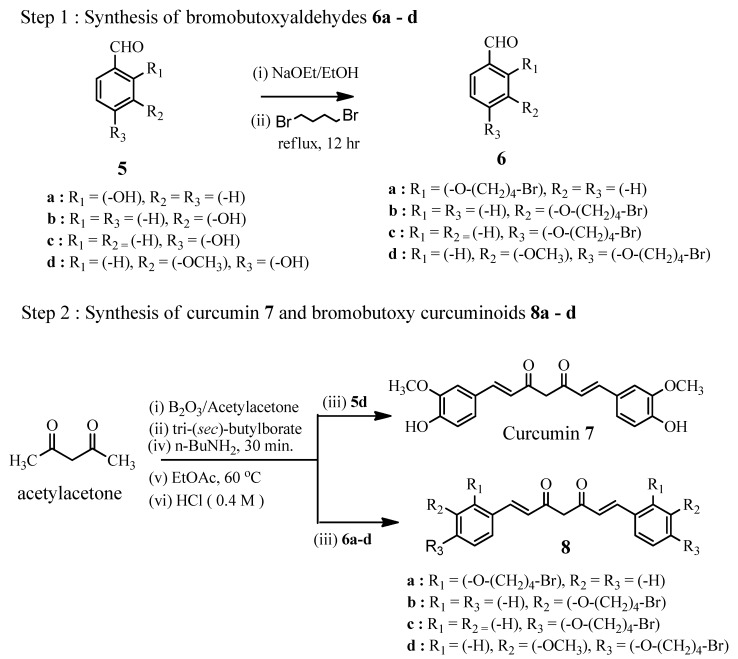

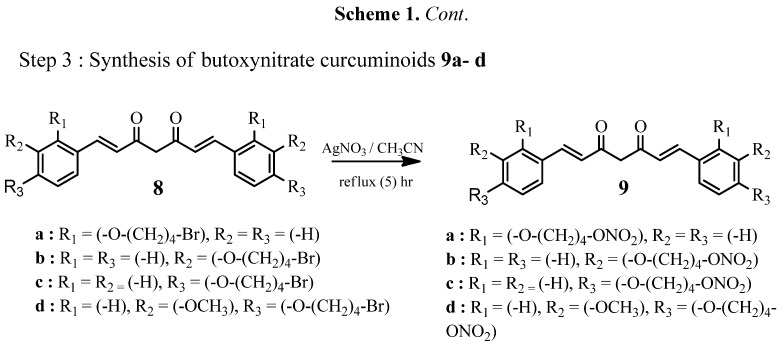
Synthesis of NO-curcuminoids **9a**–**d**.

The NO-curcuminoids were synthesized in three steps as shown in Scheme 1. The synthesis of curcuminoids from the aldehydes **5a**–**d** followed by alkylation with excess dibromobutane to produce the 4-bromobutoxy curcuminoids failed, and the best method for making the target compounds **9a**–**d** was that shown in Scheme 1. It is important to note that the synthesised NO-curcuminoids **9a**–**d** are phenolic ethers of NO as compared with the carboxylate esters of conventional NO-NSAIDs reported in the literature and shown in Figure 1. We have recently reported that 2-methoxycurcuminoid has higher anti-cancer activity compared with other derivatives [25] and in this study we have made a systematic attempt to examine the activity profile of butoxynitrate group in positions 2, 3 and 4 of the aromatic ring in curcuminoids **9a**–**d**.

All the synthesized compounds (Figure 2 and Scheme 1) were purified by flash column chromatograpy and characterized spectroscopically. Thiophene and furan curcuminoids possess potent anti-inflammatory properties [8], and the pharmacological activities of these curcuminoids was compared with our NO-curcuminoids **9a**–**d** and the natural curcumin (**7**). 

### 2.2. Pharmacology

Curcumin (**7**), its nitric oxide donating derivatives **9a**–**d** and thiophene derivatives **10a**–**d**, **11** were evaluated for cytotoxicity, production of NO and pro-inflammatory cytokines (IL-1β, TNF-α and chemokine CXCL-8) using the human monocytic leukamia, THP-1 and the human Caucasian colon adenocarcinoma-2, CACO-2 cell lines. For stimulating the cells we used the two activating agents LPS and L-methionine sulfoximine (MS). LPS acts by binding to receptors on cell membranes whilst MS acts as a membrane active agent that opens up cell membranes to release enzymes. Our aim was to compare the effects of the active compounds with the parent compound, curcumin (**7**), and to determine the structure-activity relationships of the active compounds as a guide to design future derivatives of curcumin with enhanced biological potency. 

#### 2.2.1. Cytotoxicity

**Figure 3:**
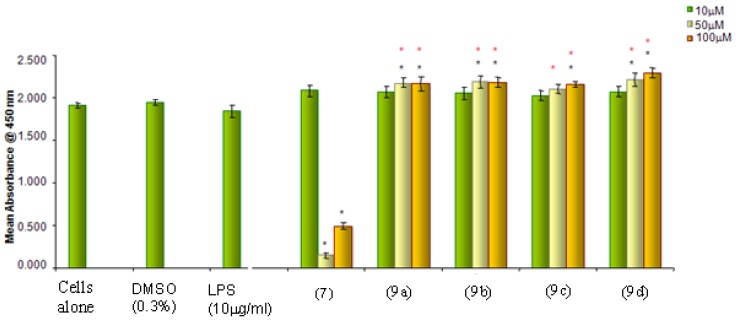
The effects of curcumin (**7**) and nitric oxide donating curcuminoids **9a**–**d** on the viability of THP-1 cells as determined by the MTS assay. Results are presented as mean ± SD. * *p* < 0.05 compared with DMSO control, * *p* > 0.05 compared with curcumin control (**7**).

At 10 µM, all compounds were as non-toxic, as was curcumin (**7**), to THP-1 cells. However, at concentrations of 50 and 100 µM, the NO donating compounds **10a**–**c** and **11** were significantly less toxic than curcumin (**7**) (Figure 3). 

From the structure-activity relationships, it appears that the replacement of both the phenolic hydrogens of curcumin (**7**) with the nitroxybutyl ether moiety enhances the non-cytotoxic properties of these compounds. However, the presence of the methoxy group (-OCH_3_) at the *meta* positions of the phenyl rings, as is found in compounds **7** and **9d**, does not seem to be crucial for the cytotoxic effects since compounds **9a**–**c** do not possess any -OCH_3_ groups and these were as non-cytotoxic as **9d** at 50 and 100 μM (Figure 3). Furthermore, change in the position of the nitroxybutyl moiety in structures of the compounds **9a**–**c** also did not have any effect on cell viability. Thus, it appears that all of the nitroxybutyl curcuminoids **9a**–**d** are non-toxic to THP-1 cells and are less cytotoxic than curcumin (**7**) at 10, 50 and 100 μM concentrations.

**Figure 4:**
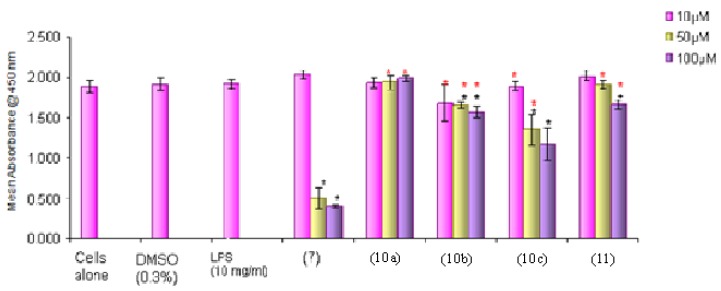
The effects of curcumin (**7**) and thiophene curcuminoids **10a**–**c**, **11** on the viability of THP-1 cells as determined by the MTS assay. Results are presented as mean ± SD. * *p* < 0.05 compared with DMSO control, * *p* > 0.05 compared with curcumin control (**7**).

**Figure 5:**
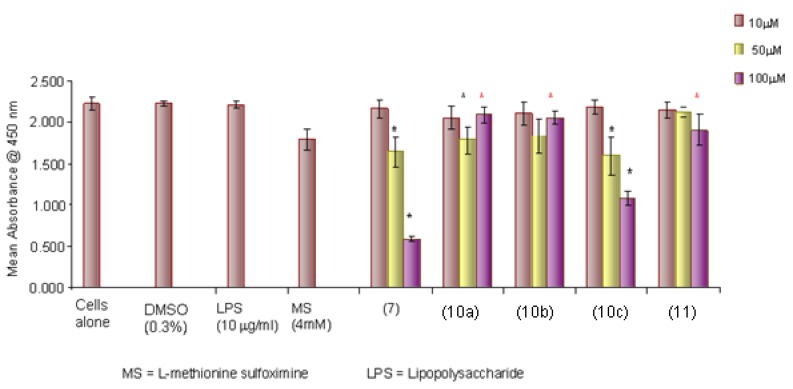
The effects of curcumin (**7**) and thiophene curcuminoids **10a**–**c**, **11** on the viability of CACO-2 cells as determined by the MTS assay. Results are presented as mean ± SD. * *p* < 0.05 compared with DMSO control, * *p* > 0.05 compared with curcumin control (**7**).

The replacement of both of the phenyl rings of the curcumin (**7**) with thiophene rings resulted in lesser cytotoxic effects on THP-1 cells. Amongst all four thiophene curcuminoids, compounds **10b** and **10c** which are the methyl substituted derivatives of **10a** at the 3 and 5 positions, respectively, show significant (*p* < 0.05) cytotoxic effects in concentration-dependent manner, suggesting the methyl group as being responsible for the cytotoxic effects (Figure 4). On the other hand compound **11** which is a positional isomer of **10a** also appeared to be less cytotoxic than **10b** and **10c**, which further confirms that there is a possibility of a methyl group being involved in the induction of cytotoxic effects associated with these derivatives. In comparison with the curcumin (**7**) the thiophene curcuminoids **10a** and **11** at 10 μM, have similar cytotoxicity to curcumin **7** whereas the methyl substituted curcuminoids **10b** and **10c** appeared to be more cytotoxic than curcumin (**7**). Since **10b** and **10c** at their lowest concentration (10 μM) are more toxic than curcumin (**7**), these could serve as potential anti-cancer drugs (Figure 4). In CACO-2 cells all the compounds **10a**–**c**, **11** had a similar toxicity profile as curcumin (**7**) but at 50 μM concentration only the 5-methyl derivative **10c** showed increased cytotoxicity that was similar to that of curcumin (**7**). The toxicity profile of the 5-methylthiophene derivative **10c** appears to be very similar in both THP-1 and CACO-2 cells (Figure 5).

#### 2.2.2. Nitric Oxide Production

**Figure 6:**
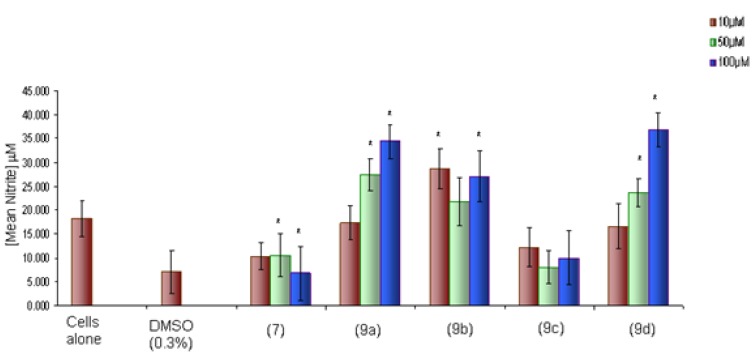
The effects of synthesized curcumin (**7**) and nitric oxide donating curcuminoids **9a**–**d** on the production of nitric oxide in THP-1 cells using the Griess reagent. Results are presented as mean ± SD. * *p* < 0.05 compared with DMSO control, * *p* > 0.05 compared with curcumin control (**7**).

**Figure 7:**
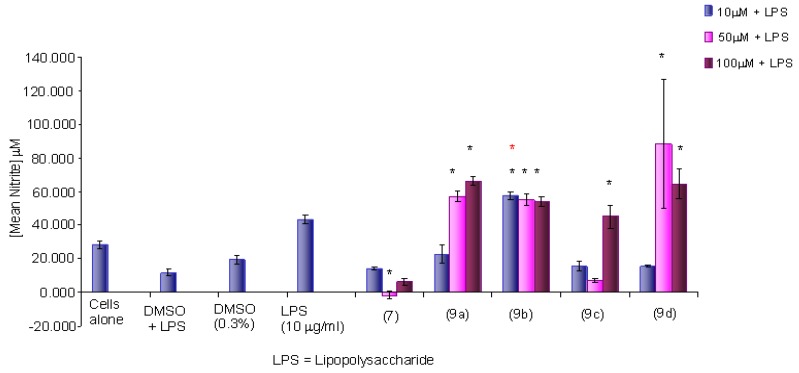
The effects of synthesized curcumin (**7**) and nitric oxide donating curcuminoids **9a**–**d** on the production of nitric oxide in LPS-induced THP-1 cells using the Griess reagent. Results are presented as mean ± SD. * *p* < 0.05 compared with DMSO + LPS control, * *p* > 0.05 compared with curcumin control (**7**).

In THP-1 cells all NO-derivatives of curcumin enhanced the production of NO in a concentration-dependent manner, except for **9c** (Figure 6 and Figure 7). From the structure activity relationships it appears that the replacement of the phenolic hydrogen of curcumin (**7**) with the nitroxybutyl ether moiety significantly enhances nitric oxide production. Compared to the DMSO control a concentration dependent increase in the production of nitrite was observed with compounds **9a**, **9b** and **9d** at all the three concentrations but the effect was significant at 50 and 100 μM. In comparison to the cells alone control, DMSO + LPS control significantly reduced the nitrite production, whereas the LPS control caused a non-significant effect in nitrite production (Figure 7). Compared with curcumin (**7**) at 10 μM concentration a significant increase in nitrite production was observed in THP-1 cells that were stimulated with LPS for compound **9b**. Compounds **9a**, **9b** and **9d** significantly increased the nitrite production at 50 and 100 μM, whereas curcumin was cytotoxic at these concentrations (Figure 7). When compared with the DMSO + LPS control a concentration dependent increase in nitrite production was observed with **9a** whilst with **9b** the effect was equally significant at all the three concentrations studied.

#### 2.2.3. Cytokine Production

**Figure 8:**
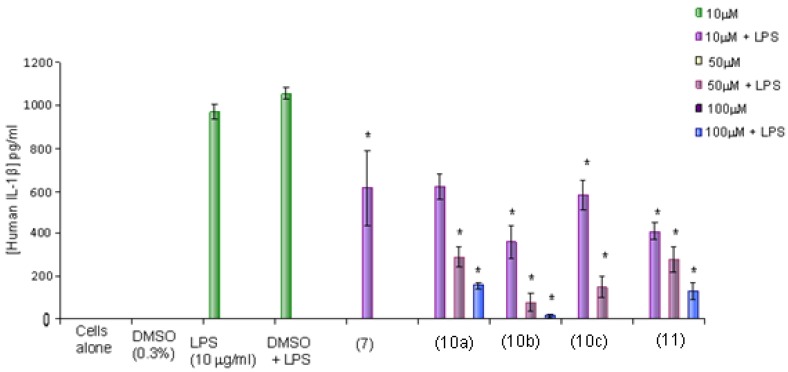
The effects of curcumin (**7**) and thiophene curcuminoids **10a**–**c**, **11** on the production of IL-1 in LPS-induced THP-1 cells using ELISA assay. Results are presented as mean ± SD. * *p* < 0.05 compared with DMSO + LPS control, * *p* > 0.05 compared with curcumin control (**7**).

The results show that curcumin (**7**) at 10 μM, **10a** and **11** significantly decreased IL-1β production in a concentration-dependent manner in THP-1 cells stimulated with LPS. In the case of compounds **10b** and **10c** the decreased effect in IL-1β production exhibited at 10 µM was also significant and this may be attributed to cytotoxicity. In comparison, all compounds were as potent as curcumin (**7**) at 10 µM. The thiophene curcuminoids **10a** from 10 to 100 μM and **11** at 10 to 50 μM range may be potential future anti-inflammatory drugs as they inhibit IL-1β production in THP-1 leukaemia cells and have low cytotoxicity (Figure 8).

**Figure 9:**
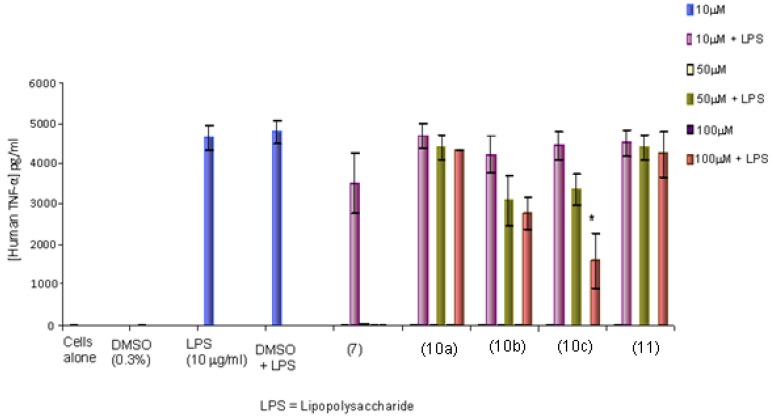
The effects of synthesized curcumin (**7**) and thiophene curcuminoids **10a**–**c**, **11** on the production of TNF-α in LPS-induced THP-1 cells using ELISA assay. Results are presented as mean ± SD. * *p* < 0.05 compared with DMSO + LPS control, * *p* > 0.05 compared with curcumin control (**7**).

Curcumin (**7**) inhibits the production of pro-inflammatory cytokines LPS-induced IL-1, IL-6 and TNF-α in THP-1 cells [20]. None of the thiophene curcuminoids **10a**–**c**, **11** or curcumin (**7**) alone affected production of TNF-α at all the three concentration 10, 50 and 100 μM. However, treatment of LPS stimulated cells with curcumin (**7**) for 24 h resulted in a concentration-dependent decrease in TNF-α production albeit the effects being significant only at 50 and 100 μM. This inhibitory effect might be due to cytotoxic effects (Figure 9). Furthermore, none of the thiophene curcuminoids **10a**–**c**, **11** at their non-cytotoxic concentrations inhibited TNF-α production in LPS stimulated cells. Curcumin (**7**) and all its synthesised thiophene derivatives **10a**–**d**, **11** appeared to be non-cytotoxic at 10 μM concentration (Figure 9). 

#### 2.2.4. Chemokine Production

**Figure 10:**
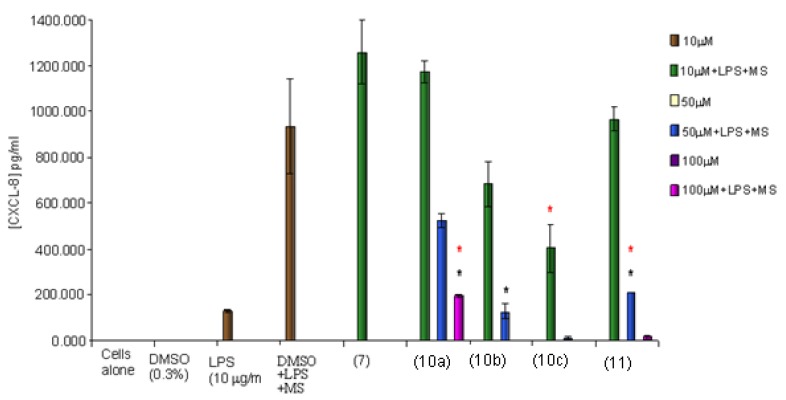
The effects of curcumin (**7**) and thiophene curcuminoids **10a**–**c**, **11** on the production of CXCL-8 in MS and LPS-induced CACO-2 cells using ELISA assay. Results are presented as mean ± SD. * *p* < 0.05 compared with DMSO control, * *p* > 0.05 compared with curcumin control (**7**).

Curcumin (**7**) did not affect the LPS-induced production of the CXCL-8 chemokine compared with the solvent control (Figure 10). However, the thiophene compounds **10a**–**c** and **11** reduced the LPS-induced production of CXCL-8 chemokine in a concentration-dependent manner compared with the DMSO solvent control. Thus at 10 µM, compounds **10b** and **10c** significantly reduced the production of CXCL-8 whereas **10a** and **11**, like curcumin (**7**), did not affect the production of CXCL-8. At 50 µM all the compounds significantly reduced the CXCL-8 production except **10c**. At the highest concentration of 100 µM, a significant decrease in CXCL-8 production was observed with all the compounds. A significant increase in the production of CXCL-8 was observed when the cells were treated with MS in the presence of LPS and DMSO. In comparison, the combined treatments with the (DMSO + LPS + MS) control, curcumin (**7**), as well as compounds **10a** and **10b** were effective inhibitors.

#### 2.2.5. Discussion of Pharmacology and Conclusions

The cytotoxicity studies showed that all of the nitroxybutyl curcuminoids **9a**–**d** and thiophene curcuminoid **10a** appeared in general to be non or less toxic to THP-1 cells than curcumin (**7**). Thus, addition of the nitrobutoxy moiety has the effect of reducing cytotoxicity of curcumin. These nitroxybutyl curcuminoids **9a**–**d** all produce nitric oxide, but this was not evident with curcumin (**7**). High concentrations of some of the nitroxybutyl curcuminoids variously reduces production of IL-1β and TNFα and in the case of thiophene compounds reduces production of the CXCL-8 chemokine. The thiophene compounds **10a**–**c**, **11** and some of the nitroxybutyl curcuminoinds **9a**–**d** may have potential in the future as anti-inflammatory agents.

## 3. Experimental

### 3.1. Chemistry: General

All apparatus was oven-dried overnight prior to use. Ethanol and ethyl acetate were dried over molecular sieves, other solvents and chemicals were used as received without further purification. ^1^H-and ^13^C- Nuclear magnetic resonance (NMR) spectra were recorded on a Bruker AC 250 spectrometer operating at 250 and 62.9 MHz, respectively, for solutions in deuterated chloroform, unless otherwise stated. Chemical shifts (δ) were recorded in parts per million (ppm) relative to the reference, Tetramethylsilane (TMS) and coupling constants (*J*) were calculated in Hz. Electron impact mass spectra (EIMS) were recorded on a VG 7070 Analytical Mass spectrometer. Electrospray mass spectra (ESMS) were obtained on Micromass platform single quadrupole mass spectrometer fitted with a Harvard syringe driver. The accurate mass of the compounds was detected using an Applied Biosystems/MDS Sciex Hybrid quadrupole time-of-flight instrument (Q-Star Pulsar-i) fitted with an orthogonal MALDI ion source and an ND:VAG Laser. Infrared spectra were recorded on ATI Mattson Genesis Series FTIR spectrophotometer using either potassium bromide pellets as a support for solid samples or sodium chloride disc for liquid samples. Melting points (°C) were recorded on Stuart SMP 3 digital Electrothermal melting point apparatus and are uncorrected. The compounds/hydroxypropyl-γ-cyclodextrin complexes were freeze-dried using a Thermo ModulyoD freeze-dryer. Curcumin (7) and curcuminoids **10a**–**c** have been previously reported [8].

#### 3.1.1. Preparation of tri-*sec*-Butyl Borate

A mixture of powdered boric acid (12.4 gm; 0.2 mol) and 2-butanol (44.4 gm; 0.6 mol) in toluene (120 mL) was refluxed with azeotropic removal of water using a Dean-Stark apparatus. The toluene was evaporated on a rotary evaporator at 60 °C to give tri-*sec*-butyl borate as a clear liquid which was stored in a tightly sealed bottle. Acetyl acetone/boron oxide complex was prepared according to the method of Pabon [26]. 

#### 3.1.2. General Procedure for the Synthesis of Bromobutoxybenzaldehydes **6a**–**d**

All four compounds **6a**–**d** were synthesized by a typical procedure which is illustrated for the formation of compound *2-(4-bromobutoxy)benzaldehyde* (**6a**). A three-neck round bottom flask fitted with a dropping-funnel, and a double surface condenser having a calcium chloride drying-tube was charged with dry EtOH (60 mL). Freshly cut sodium metal (2.3 gm; 0.1 mol) pre-washed in toluene was added slowly to EtOH with gentle stirring under reflux, until all the sodium had reacted. 2-Hydroxybenzaldehyde (0.1 mol) was added and the reaction mixture was heated at 80 °C for 30 min. 1, 4-Dibromobutane (65 gm; 0.3 mol) was added dropwise to the reaction mixture through a dropping funnel over a period of 35 min. The reaction mixture was refluxed for 12 h after which the mixture was allowed to settle and then filtered by suction filtration on a Buchner flask. To the filtrate was added water (60 mL) and extracted with EtOAc (2 × 60 mL). The organic layers were combined and dried over MgSO_4_, filtered and the solvent was evaporated on a rotary evaporator. The residue was distilled under reduced pressure (B.P. 45–50 °C/8mmHg) to remove the excess 1,4-dibromobutane and the crude product was purified by silica-gel flash chromatography using [pet. Ether–EtOAc (8:1 v/v)] as eluent, to yield product **2a**, (58%) as a light yellow oil, *R*_f_ 0.45 [pet. ether-EtOAc, 5:1 v/v]. IR (ν) 3075 (aromatic C-H stretch), 2932 (aliphatic C-H stretch), 2759 (aldehyde C-H stretch), 1687 (conjugated >C=O stretch), 1598 and 1577 (aromatic C=C stretch), 1242 (asymmetric C-O-C stretch), 1042 (symmetric C-O-C stretch), 758 cm^−1^ (*ortho* di-substituted out of plane C-H stretch); ^1^H-NMR δ 1.89–2.17 (4H, m, -CH_2_-CH_2_-), 3.50 (2H, t, *J* = 6.2 Hz, -CH_2_-Br), 4.11 (2H, t, *J* = 5.6 Hz, -O-CH_2_-), 6.95–7.05 (2H, m, Ar H-3 and H-5), 7.53 (1H, dt, *J* = 6.9 and 1.5 Hz, Ar H-4), 7.82 (1H, dd, *J* = 7.7 and 1.5 Hz, Ar H-6), 10.5 (1H, s, -CHO); EIMS *m/z* 256 [M ^79^Br]^+^ (12%), 258 [M ^81^Br]^+.^ (12%), 227 [M−CHO]^+.^ (3%), 135 [C_4_H_8_^79^Br]^+^ (62%), 137 [C_4_H_8_^81^Br]^+^ (59%), 121 [C_7_H_5_O_2_]^+^ (85%); Accurate mass found: *m/z* 256.0093 (Br ^79^), calculated for C_11_H_13_O_2_^79^Br: 256.0099.

*3-(4-Bromobutoxy)benzaldehyde* (**6b**). Yield: 79%, as a pale yellow oil, *R*_f_ 0.50 [pet. Ether–EtOAc, 5:1 v/v]. IR (ν) 3067 (aromatic C-H stretch), 2945 (aliphatic C-H stretch), 2729 (aldehyde C-H stretch), 1696 (conjugated C=O stretch), 1596 and 1585 (aromatic C=C stretch), 1262 (asymmetric C-O-C stretch), 1043 cm^−1^ (symmetric C-O-C stretch); ^1^H-NMR δ 1.90–2.16 (4H, m, -CH_2_-CH_2_-), 3.48 (2H, t, *J* = 6.4 Hz, -CH_2_-Br), 4.04 (2H, t, *J* = 5.9 Hz, -O-CH_2_-), 7.11–7.46 (4H, m, Ar H), 9.95 (1H, s, -CHO); EIMS *m/z*: 256 [M ^79^Br]^+.^ (7%), 258 [M ^81^Br]^+.^ (7%), 135 [C_4_H_8_^79^Br]^+^ (71%), 137 [C_4_H_8_^81^Br]^+^ (69%), 121 [C_7_H_5_O_2_]^+^ (69%); Accurate mass found: m/z 256.0089, calculated for C_11_H_13_O_2_^79^Br: 256.0099. 

*4-(4-Bromobutoxy)benzaldehyde* (**6c**). Yield: 81%, as golden yellow oil, *R*_f_ 0.47 [pet. Ether–EtOAc, 5:1 v/v]. IR (ν) 3074 (aromatic C-H stretch), 2945 (aliphatic C-H stretch), 2738 (aldehyde C-H stretch), 1690 (conjugated C=O stretch), 1600 and 1577 (aromatic C=C stretch), 1255 (asymmetric C-O-C stretch), 1040 (symmetric C-O-C stretch), 832 cm^−1^ (*para* disubstituted out of plane C-H stretch); ^1^H-NMR δ 1.85–2.13 (4H, m, -CH_2_-CH_2_-), 3.49 (2H , t, *J* = 6.4 Hz, -CH_2_-Br), 4.08 (2H, t, *J* = 5.9 Hz, -O-CH_2_-), 6.98 (2H, d, *J* = 8.8 Hz, Ar H-3 and H-3’), 7.82 (2H, d, *J* = 8.3 Hz, Ar H-2 and H-2’), 9.87 (1H, s, -CHO); EIMS *m/z* 256 [M ^79^Br]^+^ (6%), 258 [M ^81^Br]^+.^ (6%), 135 [C_4_H_8_^79^Br]^+^ (58%), 137 [C_4_H_8_^81^Br]^+^ (56%); Accurate mass found: *m/z* 257.0176, calculated for C_12_H_14_O_2_^79^Br: 257.0171.

*4-(4-Bromobutoxy)-3-methoxybenzaldehyde* (**6d**). Yield: 50%, as a white solid, *R*_f_ 0.33 [pet. Ether–EtOAc, 3:1 v/v], m.p. 49.7 °C. IR (ν) 3081 (aromatic C-H stretch), 2872 and 2933 (aliphatic C-H stretch), 2756 and 2821 (aldehyde C-H stretch), 1679 (conjugated C=O stretch), 1596 and 1584 (aromatic C=C stretch), 1263 (asymmetric C-O-C stretch), 1046 cm^−1^ (symmetric C-O-C stretch); ^1^H-NMR δ 2.06–2.08 (4H, m, -CH_2_-CH_2_-), 3.51 (2H, t, *J* = 6.4 Hz, -CH_2_-Br), 3.92 (3H, s, -O-CH_3_), 4.14 (2H, t, *J* = 5.9 Hz, -O-CH_2_-), 6.96 (1H, d, *J* = 7.7 Hz, Ar H-5), 7.40–7.46 (2H, m, Ar H-2 and H-6), 9.85 (1H, s, -CHO); EIMS *m/z* 286 [M ^79^Br]^+.^ (9%), 288 [M ^81^Br]^+.^ (8%), 135 [C_4_H_8_^79^Br]^+^ (54%), 151 [C_4_H_8_^81^Br]^+^ (54%); Accurate mass found: *m/z* 286.0200 (^79^Br), calculated for C_12_H_15_O_3_^79^Br: 286.0205.

#### 3.1.3. Synthesis of Curcuminoids

*Method A*: In this procedure the synthesis of curcuminoids was carried out according to Pabon [8,21] in which separately prepared acetyl acetone-boron oxide complex was used.

*Method B*: In this procedure the synthesis of curcuminoids was carried out by the *in-situ* formation of acetyl-acetone-boron oxide complex.

The crude products **8a**–**d** obtained were all purified by flash column chromatography using [pet. Ether-EtOAc 8:1 v/v] as eluent.

*(1E,4Z,6E)-1,7-Bis(2-94-bromobutoxy)phenyl)-5-hydroxyhepta-1,4,6-trien-3-one* (**8a**). Yield: 34% (method A), 21% (method B), a dark brown gum, *R*_f_ 0.44 [pet. ether-EtOAc, 3:1 v/v]. IR (KBr pellet) ν 3032 (aromatic C-H stretch), 2924 (aliphatic C-H stretch), 1620 (H-bonded >C=O stretch), 1595 and 1570 (aromatic C=C stretch), 1244 (asymmetric C-O-C stretch), 1046 (symmetric C-O-C stretch), 750 cm^−1^ (*ortho* disubstituted out of plane C-H stretch); ^1^H-NMR δ 1.98–2.20, (8H, m, -CH_2_-CH_2_-), 3.55 (4H, t, *J* = 5.9 Hz, -CH_2_-Br), 4.10 (4H, t, *J* = 5.6 Hz, -O-CH_2_-), 5.87 (1H, s, enol H), 6.75 (2H, d, *J* = 16.0 Hz, Ar-CH=CH-), 6.91 (2H, d, *J* = 8.2 Hz, Ar H-3), 6.98 (2H, t, *J* = 7.7 Hz, Ar H-5), 7.33 (2H, dt, *J* = 8.7 and *J* = 1.7 Hz, Ar H-4), 7.57 (2H, dd, *J* = 7.7 and *J* = 1.5 Hz, Ar H-6), 7.97 (2H, d, *J* = 16.0 Hz, -CO-CH=CH-Ar); ^13^C-NMR δ 28.1, 29.8, 33.7, 67.7, 101.9, 112.4, 121.2, 124.5, 125.2, 129.1, 131.5, 136.0, 157.9, 184.0; EIMS *m/z* 576 [M ^79^Br]^+.^ (4%), 580 [M ^81^Br]^+.^ (6%), 577 [M+H]^+.^, (2%), 578, [M+2H]^+.^, (7%), 135 [C_4_H_8_^79^Br]^+^ (39%), 137 [C_4_H_8_^81^Br]^+^ (37%); Accurate mass found: *m/z* 577.0583, calculated for C_27_H_31_O_4_^79^Br_2_: 577.0589. 

*(1E,4Z,6E)-1,7-Bis(3-(4-bromobutoxy)phenyl)hepta-1,6-dien-3,5-dione* (**8b**). Yield: 24% (method A) 21% (method B), as a dark brown solid, *R*_f_ 0.41 [pet. ether-EtOAc, 3:1 v/v], m.p. 74.6–75.9 °C. IR (ν) 3426 (OH stretch), 3052 (aromatic C-H stretch), 2947 (aliphatic C-H stretch), 1625 (H-bonded >C=O stretch), 1596 and 1579 (aromatic C=C stretch), 1508 (enol), 1243 (asymmetric C-O-C stretch), 1044 (symmetric C-O-C stretch), 885, 791, 677 cm^−1^ (*meta* disubstituted out of plane C-H stretch); ^1^H-NMR δ 1.95–2.18 (8H, m, -CH_2_-CH_2_-), 3.51 (4H, t, *J* = 6.45 Hz, -CH_2_-Br), 4.04 (4H, t, *J* = 5.9 Hz, -O-CH_2_-), 5.85 (1H, s, enol H), 6.62 (2H, d, *J* = 16.0 Hz, Ar-CH=CH-), 6.92 (2H, d, *J* = 7.7 Hz, Ar H-4), 7.07 (2H, s, Ar H-2), 7.16 (2H, d, *J* = 7.7 Hz, Ar H-6), 7.31 (2H, t, *J* = 7.9 Hz, Ar H-5), 7.63 (2H, d, *J* = 16.0 Hz, -CO-CH=CH-Ar); ^13^C-NMR δ 28.1, 29.8, 33.6, 67.2, 102.0, 113.9, 116.7, 121.2, 124.7, 130.2, 136.7, 140.8, 159.5, 183.5; ESMS *m/z* 576 [M ^79^Br]^+.^, 577 [M ^79^Br+H]^+.^, 578 [M ^79^Br+2H]^+.^, 580 [M ^81^Br]^+.^, 581 [M ^81^Br+H]^+.^, 582, [M ^81^Br+2H]^+.^.

*(1E,6E)-1,7-Bis(4-(4-bromobutoxy)phenyl)hepta-1,6-dien-3,5-dione* (**8c**). Yield: 66% (method A), 41% (method B), as a bright yellow solid, *R*_f_ 0.42 [pet. ether-EtOAc, 3:1 v/v], m.p. 145–146.9 °C. IR (ν) 3433 (OH stretch), 3035 (aromatic C-H stretch), 2945 (aliphatic C-H stretch), 1628 (H-bonded >C=O stretch), 1600 (aromatic C=C stretch), 1510 (enol), 1420 (olefinic in plane bending vibration), 1257 (asymmetric C-O-C stretch), 1046 (symmetric C-O-C stretch), 837 cm^−1^ (*para* disubstituted out of plane C-H stretch); ^1^H NMR δ 1.92–2.14 (8H, m, -CH_2_-CH_2_-), 3.50 (4H, t, *J* = 6.4 Hz, -CH_2_-Br), 4.04 (4H, t, *J* = 5.6 Hz, -O-CH_2_-), 5.78 (1H, s, enolic CH=C), 6.50 (2H, d, *J* = 15.5 Hz, Ar-CH=CH-), 6.90 (4H d, *J* = 8.8 Hz, Ar H-3 and H3’), 7.50 (4H, d, *J* = 8.7 Hz, Ar H-2 and H2’), 7.62 (2H, d, *J* = 15.5 Hz, -CO-CH=CH-Ar); ^13^C-NMR δ 29.7, 28.1, 67.3, 101.6, 115.1, 122.1, 128.3, 130.0, 140.3, 160.8, 183.6; EIMS *m/z* 576 [M ^79^Br]^+^ (3%), 578 [M ^79^Br+2H]^+^ (8%), 135, [C_4_H_8_^79^Br]^+^(100%), 137 [C_4_H_8_^81^Br]^+^ (98%); Accurate mass found: *m/z* 576.0535, calculated for C_27_H_30_O_4_^79^Br_2_ : 576.0511). 

*(1E,4Z,6E)-1,7-Bis(4-(4-bromobutoxy)-3-methoxyphenyl)hepta-1,6-dien-3,5-dione* (**8d**). Yield: 57% (method A), as a dark yellow solid between EtOAc and aqueous layers during work-up and no further purification was required; *R*_f_ 0.38 [pet. ether-EtOAc, 2:1 v/v], m.p. 124.4–125.4 °C. IR (ν) 3548-3235 (OH enolic), 3003 (aromatic C-H stretch), 2955 and 2870 (aliphatic C-H stretch), 1620 (H-bonded >C=O), 1597 and 1581 (aromatic C=C stretch), 1508 (enol), 1422 cm^−1^ (olefinic in plane bending vibration); ^1^H-NMR δ 2.00–2.17 (8H, m, -CH_2_-CH_2_-), 3.51 (4H t, *J* = 6.4 Hz, -CH_2_-Br), 3.92 (6H, s, -OCH_3_), 4.10 (4H, t, *J* = 5.9 Hz, -O-CH_2_-), 5.82 (1H, s, enol H), 6.50 (2H, d, *J* = 16.0 Hz, Ar-CH=CH-), 6.87 (2H, d, *J* = 8.2 Hz, Ar H-5), 7.08 (2H, d, *J* = 2.0 Hz, Ar H-2), 7.13 (2H, dd, *J*_1,3_ = 8.2 and *J*_1,2_ = 1.5 Hz, Ar H-6), 7.61 (2H, d, *J* = 15.5 Hz, -CO-CH=CH-Ar); EIMS *m/z* 637 [M+H]^+^ (18%), 619 [M+H−H_2_O]^+^ (31%), 324 [C_15_H_18_O_3_^79^Br+H]^+^ (31%), 326 [C_15_H_18_O_3_^81^Br+H]^+^ (29%), 324 [C_14_H_16_O_3_^79^Br]^+^ (20%), 313 [C_14_H_16_O_3_^81^Br]^+^ (18%), 135 [C_4_H_8_^79^Br]^+^ (100%), 137 [C_4_H_8_^81^Br]^+^ (99%); Accurate mass found: *m/z* 636.0714 (Br^79^), calculated for C_29_H_34_O_6_^79^Br_2_ : 636.0722.

#### 3.1.4. Synthesis of Nitroxybutyl Curcuminoids **9a**–**d**

The general procedure for the synthesis of butoxy-nitrate curcuminoids **9a**–**d** is illustrated for the formation of *(1E,6E)-1,7-bis(2-(4-butoxynitrato)hepta-1,6-diene-3,5-dione* (**9a**). In a one-neck round bottom flask, a mixture of silver nitrate (1.17 gm, 6.9 mmol) in acetonitrile (3 mL) was stirred for 30 min. and then a solution of **8a** (0.86 mmol) in acetonitrile (2 mL) was added. The reaction mixture was refluxed under stirring for 5 h at 80 °C. An aluminium sheet was wrapped round the flask to protect it from light and the mixture was allowed to stand overnight. Water (5 mL) was added and after filtration the mixture was extracted with EtOAc (2 × 30 mL). The organic layers were combined, dried over MgSO_4_, filtered under gravity and the solvent was removed on the rotary evaporator to yield pure **9a** (90%) as a dark brown gum, *R*_f_ 0.43 [pet. ether-EtOAc, 2:1 v/v], m.p. 136.0–136.8 °C. IR (KBr pellet) ν 3035 (aromatic C-H stretch), 2926 and 2878 (aliphatic C-H stretch), 1625 (conjugated >C=O stretch), 1596 and 1488 (aromatic C=C stretch), 1472 and 1280 (aliphatic NO_2_ stretch), 1455 (CH_2_ bending absorption), 1244 (asymmetric C-O-C stretch), 1049 (symmetric C-O-C stretch), 753 cm^−1^ (*ortho* disubstituted out of plane C-H stretch); ^1^H-NMR δ 1.99–2.04 (8H, m, -CH_2_-CH_2_-), 4.10 (4H, t, *J* = 5.4 Hz, -O-CH_2_-), 4.58 (4H, t, *J* = 5.9 Hz, -CH_2_-ONO_2_), 5.83 (1H, s, enol H), 6.74 (2H, d, *J* = 16.0 Hz, Ar-CH=CH-), 6.91 (2H, d, *J* = 8.2 Hz, Ar H-3), 6.99 (2H, t, *J* = 7.4 Hz, Ar H-7.97 (2H, d, *J* = 16.0 Hz, -CO-CH=CH-Ar); ^13^C-NMR δ 24.2, 25.9, 67.8, 73.1, 102.1, 112.4, 121.3, 124.6, 125.2, 129.0, 131.5, 135.8, 157.7, 183.9; ESMS *m/z* 543 [M+H]^+.^, 332 [C_17_H_18_O_6_N]^+^; Accurate mass found: *m/z* 543.1964, calculated for C_27_H_31_N_2_O_10_: 543.1973. 

*(1E,6E)-1,7-Bis(3-(4-butoxy-nitrate)hepta-1,6-diene-3,5-dione* (**9b**). Yield: 58% as a dark brown solid, *R*_f_ 0.46 [pet. ether-EtOAc, 2:1 v/v], m.p. 133.9–134.9 °C. IR (ν) 3414 (OH stretch), 3020 (aromatic C-H stretch), 2929 and 2875 (aliphatic C-H stretch), 1638 (conjugated >C=O stretch), 1596 and 1489 (aromatic C=C stretch), 1473 and 1279 (aliphatic NO_2_ stretch), 1458 (CH_2_ bending absorption), 1247 (asymmetric C-O-C stretch), 1041 cm^−1^ (symmetric C-O-C stretch); ^1^H-NMR δ 1.95 (8Hz, t, *J* = 2.8 Hz, -CH_2_-CH_2_-), 4.04 (4H, t, *J* = 5.1 Hz, -O-CH_2_-), 4.56 (4H, t, *J* = 5.9 Hz, -CH_2_-ONO_2_), 5.85 (1H, s, enol H), 6.61 (2H, d, *J* = 16.0 Hz, Ar-CH=CH-), 6.91 (2H, dd, *J* = 7.9 and 1.7 Hz, Ar H-4), 7.06 (2H, s, Ar H-2), 7.16 (2H, d, *J* = 7.7 Hz, Ar H-6), 7.31 (2H, t, *J* = 7.7 Hz, Ar H-5), 7.62 (2H, d, *J* = 15.5 Hz, -CO-CH=CH-Ar); ^13^C-NMR: δ 24.2, 25.9, 67.3, 73.1, 102.1, 113.9, 116.6, 121.3, 124.7, 130.3, 136.7, 140.7, 159.4, 183.5; ESMS *m/z* 543 [M+H]^+.^, 565 [M+Na]^+^; Accurate mass found: *m/z* 543.1985, calculated for C_27_H_31_N_2_O_10_: 543.1973. 

*(1E,6E)-1,7-Bis(4-(4-Butoxy-nitrate)hepta-1,6-diene-3,5-dione* (**9c**). Yield: 95%, golden yellow solid, *R*_f_ 0.35 (pet. ether-EtOAc, 2:1 v/v), m.p. 112.6–113 °C. IR (ν) 3412 (OH stretch), 3040 (aromatic C-H stretch), 2936 (aliphatic C-H stretch), 1620 (conjugated >C=O stretch), 1603 and 1511 (aromatic C=C stretch), 1472 and 1287 (aliphatic NO_2_ stretch), 1256 (asymmetric C-O-C stretch), 1056 (symmetric C-O-C stretch), 837 cm^−1^ (*para* disubstituted out of plane C-H stretch); ^1^H-NMR δ 1.95 (8H, t, *J* = 2.2 Hz, -CH_2_-CH_2_-), 4.06 (4H, t, *J* = 5.6 Hz, -O-CH_2_-), 4.56 (4H, t, *J* = 5.3 Hz, -CH_2_-ONO_2_), 5.79 (1H, s, enol H), 6.51 (2H, d, *J* = 15.5 Hz, Ar-CH=CH-), 6.90 (4H, d, *J* = 8.8 Hz, Ar H-3 and H-3’), 7.51 (4H, d, *J* = 8.8 Hz, Ar H-2 and H-2’), 7.63 (2H, d, *J* = 16.0 Hz, -CO-CH=CH-Ar); ^13^C-NMR: δ 24.1, 25.8, 67.4, 73.1, 101.6, 115.1, 122.2, 128.3, 130.0, 140.3, 160.7, 183.6; EIMS *m/z* 543 [M+H]^+^, (42%). 

*(1E,6E)-1,7-Bis(4-(4-butoxy-nitrate)-3-methoxyphenyl)hepta-1,6-diene-3,5-dione* (**9d**). Yield: 92% as an orange solid, *R*_f_ 0.43 [pet. ether-EtOAc, 1:3 v/v], m.p. 101.6–102.6 °C. IR (ν) 3436 (OH stretch), 2954 (aromatic C-H stretch), 2931 and 2873 (aliphatic C-H stretch), 1621 (conjugated >C=O stretch), 1458 (CH_2_ bending absorption), 1512 (aromatic C=C stretch), 1257 (asymmetric C-O-C stretch), 1027 (symmetric C-O-C stretch). ^1^H-NMR δ 1.63 (8H, s, br. -CH_2_-CH_2_-), 3.91 (6H, s, -O-CH_3_), 4.10 (4H, t, *J* = 5.1 Hz, -O-CH_2_-), 4.58 (4H, t, *J* = 5.9 Hz, -CH_2_-ONO_2_), 5.83 (1H, s, enol H), 6.50 (2H, d, *J* = 15.5 Hz, Ar-CH=CH-), 6.87 (2H, d, *J* = 8.2 Hz, Ar H-5), 7.09 (2H, s, Ar H-2 ), 7.13 (2H, d, *J* = 8.2 Hz, Ar H-6), 7.61 (2H, d, *J* = 15.5 Hz, -CO-CH=CH-Ar); EIMS *m/z* 603 [M]^+^ (38%), 604 [M+H]^+.^, (12%); Accurate mass found: *m/z* 603.2162, calculated for C_29_H_35_N_2_O_10_: 603.2184.

*1,7-Bis(thiophen-3-yl-)-1,6-heptadien-3,5,dione* (**11**). Yield: 36% (method A) purified by column chromatography (pet. ether-EtOAc, 6:1 v/v), 23% yield (method B), yellow solid, *R*_f_ 0.48 (pet. ether- EtOAc, 5:1 v/v), m.p. 140.1–141.3 °C.; IR (ν) 3435 (OH stretch), 3092 (aromatic C-H stretch), 1624 (H-bonded >C=O), 1584 (conjugated C=C), 1507 (enol), 1412 cm^−1^ (olefinic in plane bending vibration); ^1^H-NMR δ 5.79 (1H, s, enol H), 6.45 (2H, d, *J* = 15.5 Hz, Ar-CH=CH-), 7.33–7.38 (4H, m, Ar H), 7.52 (2H, d, *J* = 2.6 Hz, Ar H-2), 7.66 (2H, d, *J* = 16.0 Hz, -CO-CH=CH-Ar); EIMS *m/z* 288 [M]^+^ (60%), 270 [M−H_2_O]^+.^ (8%), 192 [C_10_H_8_O_2_S]^+^ (4%), 179 [C_9_H_7_O_2_S]^+^ (5%), 151 [C_8_H_7_OS]^+^ (21%), 137 [C_7_H_5_OS]^+^ (100%), 109 [C_6_H_5_S]^+^ (43%), 97 [C_5_H_4_S]^+^ (21%); Accurate mass found: *m/z* 288.0277, calculated for C_15_H_12_O_2_S_2_: 288.0279. 

### 3.2. Pharmacology

#### 3.2.1. General Procedures for Cell Cultures And Compound Preparations 

Human monocytic leukemia (THP-1) cell line and human caucasian colon adenocarcinoma (CACO-2) cell lines were obtained from the European Collection of Cell Cultures (ECACC) (Salisbury, Wiltshire, UK). Preparation of drug treatments and general procedures for cell culture were as previously described [27,28,29,30]. All cell culture techniques, *i.e.*, thawing, passaging, plating as well as respective compound treatments applied to the cells and cytotoxicity using 3-(4,5-dimethylthiazol-2yl)-5-(3-carboxymethoxyphenyl)-2-(4-sulfophenyl)-2H-tetrazolium, inner salt (MTS) assays were performed under sterile conditions using a laminar flow cabinet. In these experiments the colorimetric assay CellTiter 96^®^ AQ_ueous_ one solution cell proliferation *in vitro* was used to examine the effects of the synthesized curcumin (**7**) and curcuminoids on cell viability [27]. For experimental work, samples were prepared in media, without serum (serum-free RPMI) for THP-1 cells and CACO-2 cells respectively. Lipopolysaccharide (LPS) *Escherichia coli* O127:B8 (cell culture tested) and L-methionine sulfoximine (MS) were obtained from Sigma-Aldrich (St. Louis, MO, USA)). All incubations were carried out in a humidified incubator maintained at 37 °C with 5% (v/v) CO_2_ and 95% air. Experiments were performed in duplicate wells of the 96-well plates and were repeated at least four times unless otherwise stated in the figure legend. 

For all MTS, nitric oxide and ELISAs, the stock solutions (33 mM) of all the compounds were prepared in DMSO (100%) followed by appropriate dilutions. For the determination of cytotoxic effects of the compounds **9a**–**d** these were complexed with hydroxypropyl-γ-cyclodextrin (HP-γ-CD) for improved solubilisation. A 100 mM stock solution was prepared using DMSO (100%). The drug/ HP-γ-CD complex (1:2 molar ratio) was diluted to 10, 50 and 100 µM (relative to the drug concentration) [27]. 

#### 3.2.2. Nitric Oxide Assay

The determination of nitrite in biological samples was measured by the spectrophotometric method using Griess reagent. NO levels are quantified by estimating its stable end product (nitrite anion) which is formed as a result of nitric oxide oxidation [31]. Experimental samples (50 µL/well) were added to duplicate wells of a 96-well plate, 50 µL of Griess reagent was then added and samples incubated for 5–10 min at room temperature protected from light for colour development. Absorbance was measured at 570 nm using a plate reader (Wallac Victor 2 multi-label plate reader). Nitrite concentrations were determined from a sodium nitrite standard curve.

#### 3.2.3. Sandwich ELISA Assay 

Sandwich ELISAs are a widely used standard method for the quantitative detection of cytokines and other specific proteins in serum samples. Substrate was added which reacted with the enzyme producing a colour change, the intensity of which is directly proportional to the concentration of antigen in incubation mixture allowing its spectrophotometric detection and quantification. Standard proteins of known concentrations are incorporated into the assay to allow quantification of specific proteins to be determined [32,33,34]. The capture antibodies, mouse anti-human IL-1β or mouse anti-human TNF-α were diluted to a working concentration of 4.0 µg/ml in PBS (without carrier proteins). ELISA flat-bottom 96-well plates were prepared according to the manufacturer’s protocol for the detection of IL-1β or TNF-α in cell supernatants of THP-1, CXCL-8 and CACO-2 cells. An ELISA flat-bottom 96-well plate was immediately coated with 100 µL/well of the diluted capture antibody, sealed and incubated overnight at room temperature. Next day, each well of the 96-well micro plate was aspirated and washed six times with wash buffer (WB, 0.05% Tween^®^ 20 in PBS). After the last wash, the plate was dried by blotting against clean paper towel, blocked by adding 300 µL/well of reagent diluent (RD) (1%).

The 96-well microtitre plate is coated with capture antibody (1st antibody) and the target protein or antigen is added. Detection antibody (2nd antibody) is added followed by the addition of enzyme, horseradish peroxidase (HRP)-linked antibody. Substrate solution containing colorimetric substrate, 3,3’,5,5’-tetramethylbenzidine (TMB) and hydrogen peroxide (H_2_O_2_) is added. TMB reacts with H_2_O_2_ in the presence of HRP enzyme to produce a water-soluble, blue coloured by-product the intensity of which is proportional to the amount of HRP activity, which in turn is related to the levels of target analyte in the experimental sample. Upon acidification with sulphuric-acid (stop solution) the colour changes from blue to yellow, enabling accurate measurement of the intensity at 450 nm using a plate-reader.
